# Supplementary material for: Neo-5,22E-Cholestadienol Derivatives from Buthus martensii Karsch and Targeted Bactericidal Action Mechanisms
Source: Molecules. 2018 Dec 26;24(1):72. doi: 10.3390/molecules24010072 (PMC6337218; doi:10.3390/molecules24010072)
Supplement: Supplementary file 1 [file molecules-24-00072-s001.pdf]

Appendix A. Supplementary data

Neo-5,22E-cholestadienol derivatives from *Buthus martensi* karsch and targeted bactericide action mechanisms

Biyu Lv[1], Weiping Yin, [1*], Jiayu Gao[1], Huaqing Liu[1], Kun Liu[1], Jie Bai[1] and Qiangqiang Yang[2]

[1]*School of Chemical Engineering and Pharmaceutics, Henan University of Science and Technology, Luoyang 471023, P.R. China.*
[2] *Life Science and Environmental Science Research Centerof Harbin University of Commerce, Harbin, Heilongjiang , P.R.China*

*Correspondence: yinwp@haust.edu.cn(W.-P. Yin); Tel.: +86-379-642-31914

To whom correspondence E-mail address: yinwp@haust.edu.cn (W.-P.Yin).

Table of Contents: **Page:**

Compound 1(QX75-5)…………………………………. S1-S7

Compound 2 (QX37-45-4)…………………………….. S1-S15

Compound 3(QX3-2-7) ………………………………… S16-S23

Contents

NMR spectra of reported compounds, HR-ESIMS and their Circular Dichromism spectra.

Figure S1.^{1}H NMR spectrum of Compound 1 in $CDCl_3$ (400 MHz).
Figure S2. ^{13}C NMR spectrum of Compound 1 in $CDCl_3$ (400 MHz).
Figure S3. DEPT spectrum of Compound 1in $CDCl_3$ (400 MHz).
Figure S4. HSQC spectrum of Compound 1 in $CDCl_3$ (400 MHz).
Figure S5. HMBC spectrum of Compound 1 in $CDCl_3$ (400 MHz).
Figure S6.HR-ESIMS spectrum of Compound 1
Figure S7 . Circular Dichromism spectra spectrum of Compound1

Figure S8. ^{1}H NMR spectrum of Compound 2 in $CDCl_3$ (400 MHz).
Figure S9. ^{13}C NMR spectrum of Compound 2 in $CDCl_3$ (400 MHz).
Figure S10. DEPT spectrum of Compound 2 in $CDCl_3$ (400 MHz).
Figure S11. ^{1}H- ^{1}H COSY spectrum of Compound 2 in $CDCl_3$ (400 MHz).
Figure S12. HSQC spectrum of Compound 2 in $CDCl_3$ (400 MHz).
Figure S13. HMBC spectrum of Compound 2 in $CDCl_3$ (400 MHz).
Figure S14.HR-ESIMS spectrum of Compound 2
Figure S15 . Circular Dichromism spectra spectrum of Compound 2

Figure S16. ^{1}H NMR spectrum of Compound 3 in $CDCl_3$ (400 MHz).
Figure S17. ^{13}C NMR spectrum of Compound 3 in $CDCl_3$ (400 MHz).
Figure S18. DEPT spectrum of Compound 3 in $CDCl_3$ (400 MHz).
Figure S19. ^{1}H- ^{1}H COSY spectrum of Compound 3 in $CDCl_3$ (400 MHz).
Figure S20. HSQC spectrum of Compound 3 in $CDCl_3$ (400 MHz).
Figure S21. HMBC spectrum of Compound 3in $CDCl_3$ (400 MHz).
Figure S22 .HR-ESIMS spectrum of Compound 3
Figure S23 . Circular Dichromism spectra spectrum of Compound 3

Compound 1(QX75-5)**.**

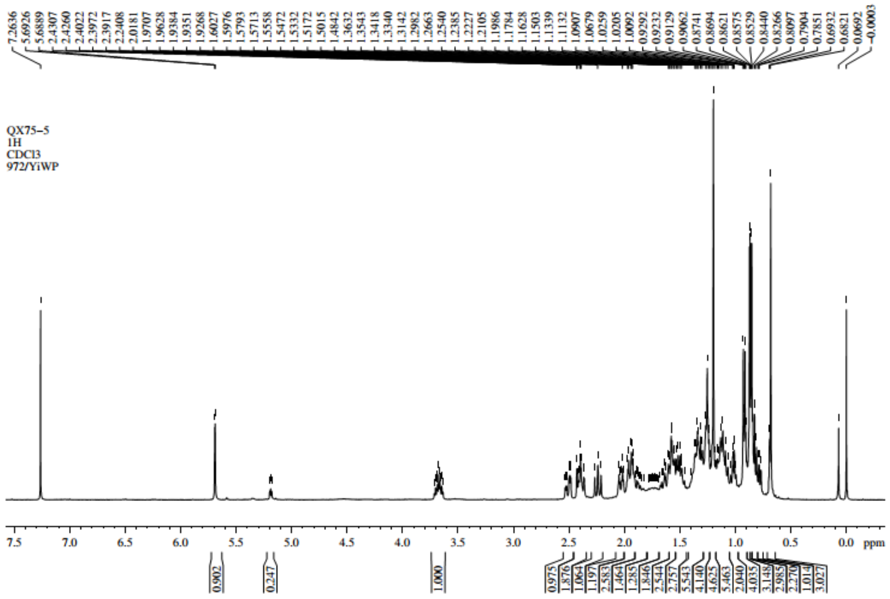

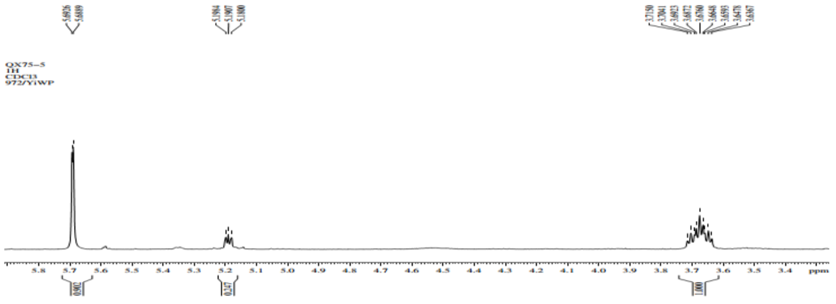

Figure S1.^{1}H NMR spectrum of Compound 1 in $CDCl_3$ (400 MHz).

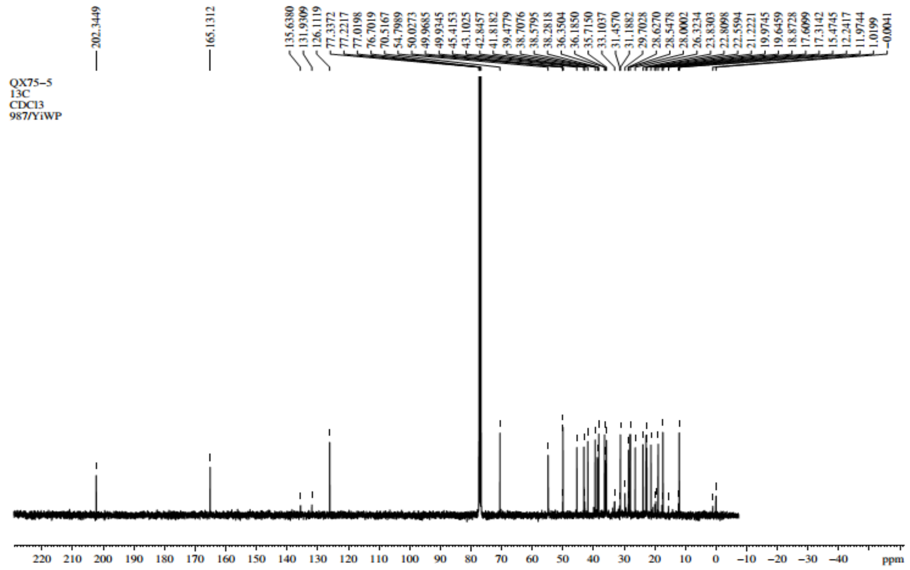

Figure S2. ^{13}C NMR spectrum of Compound 1 in $CDCl_3$ (400 MHz).

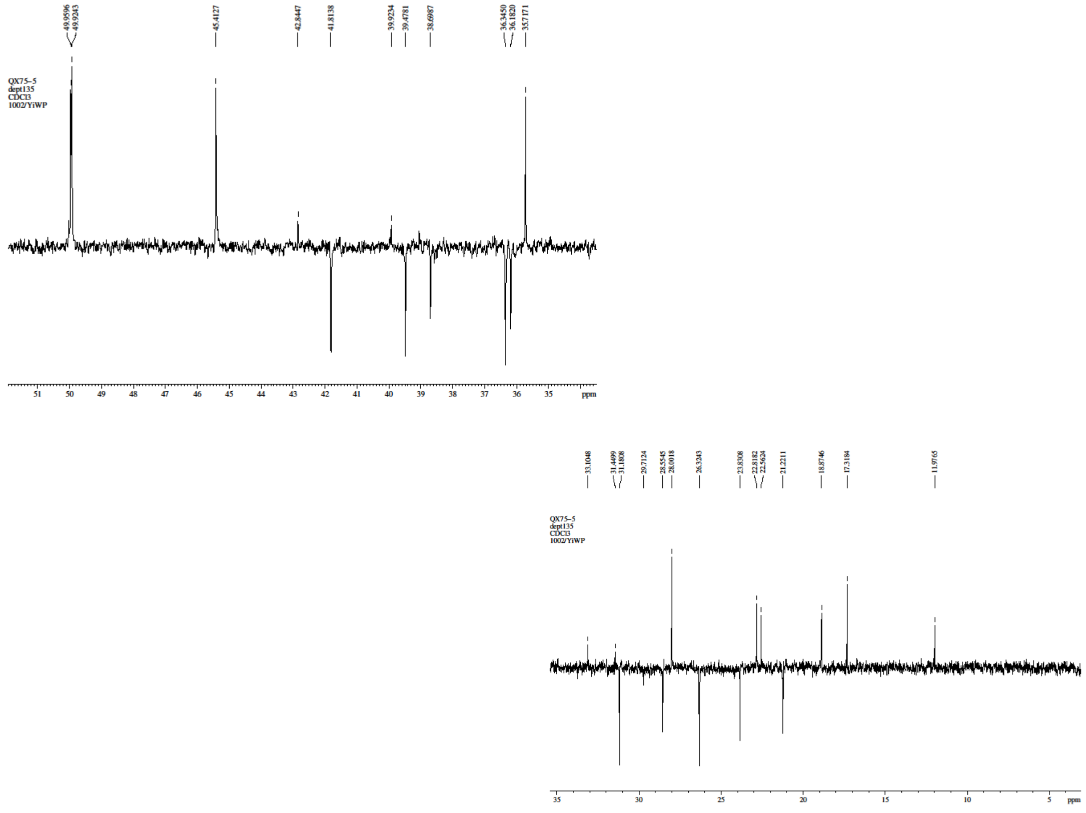

Figure S3. DEPT spectrum of Compound 1 in $CDCl_3$ (400 MHz).

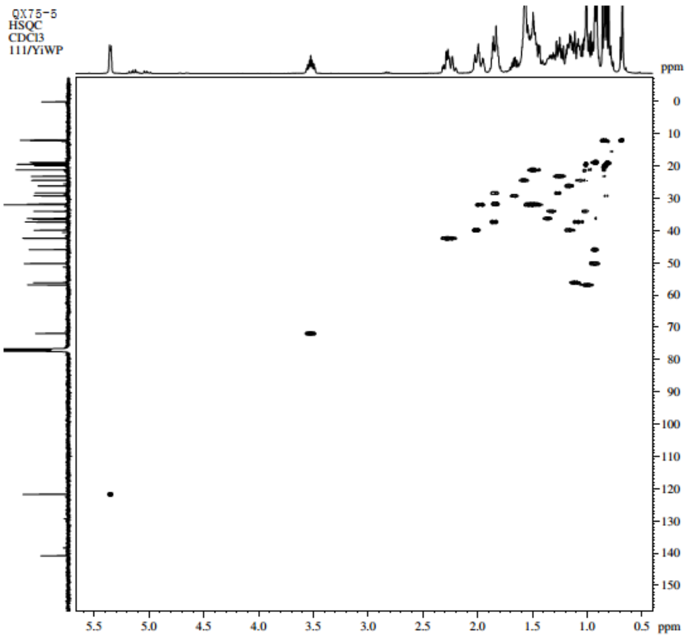

Figure S4. HSQC spectrum of Compound 1 in $CDCl_3$ (400 MHz).

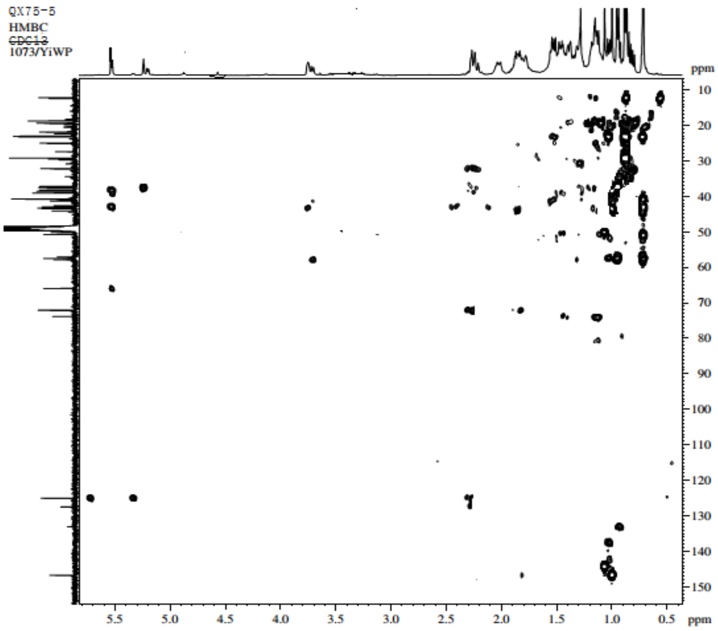

Figure S5. HMBC spectrum of Compound 1 in $CDCl_3$ (400 MHz)

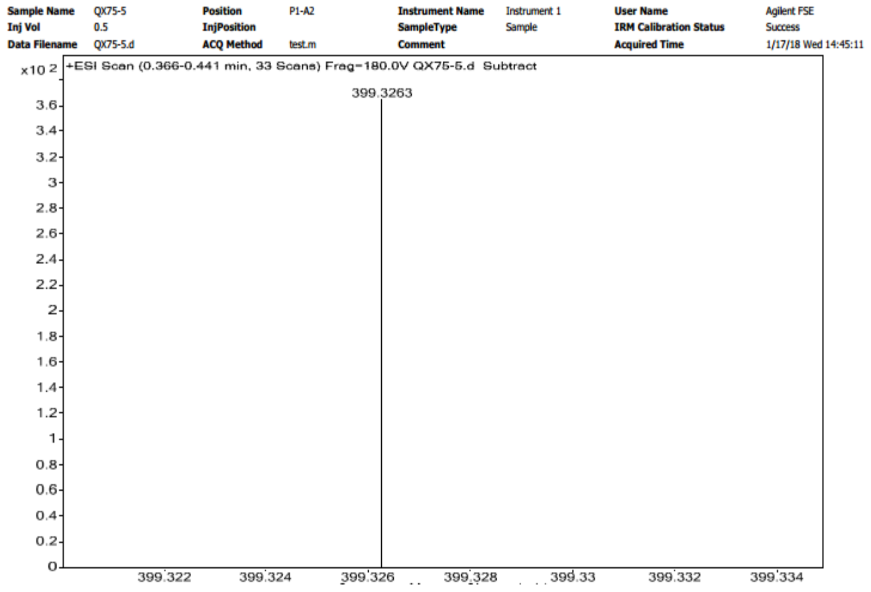

PDF created with pdfFactory trial version www.pdffactory.com

Figure S6.HR-ESIMS spectrum of Compound 1

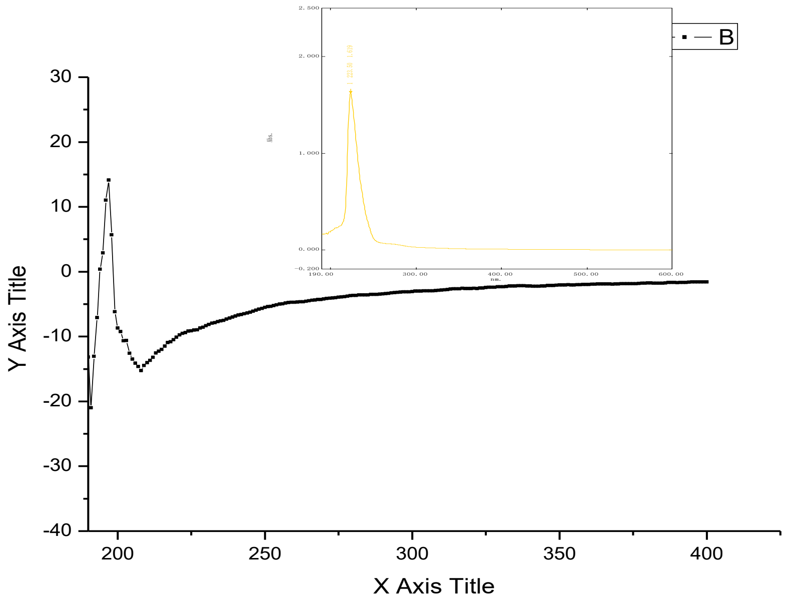

Figure S7 . Circular Dichromism spectra spectrum of Compound 1

Compound 2 (QX37-45-4).

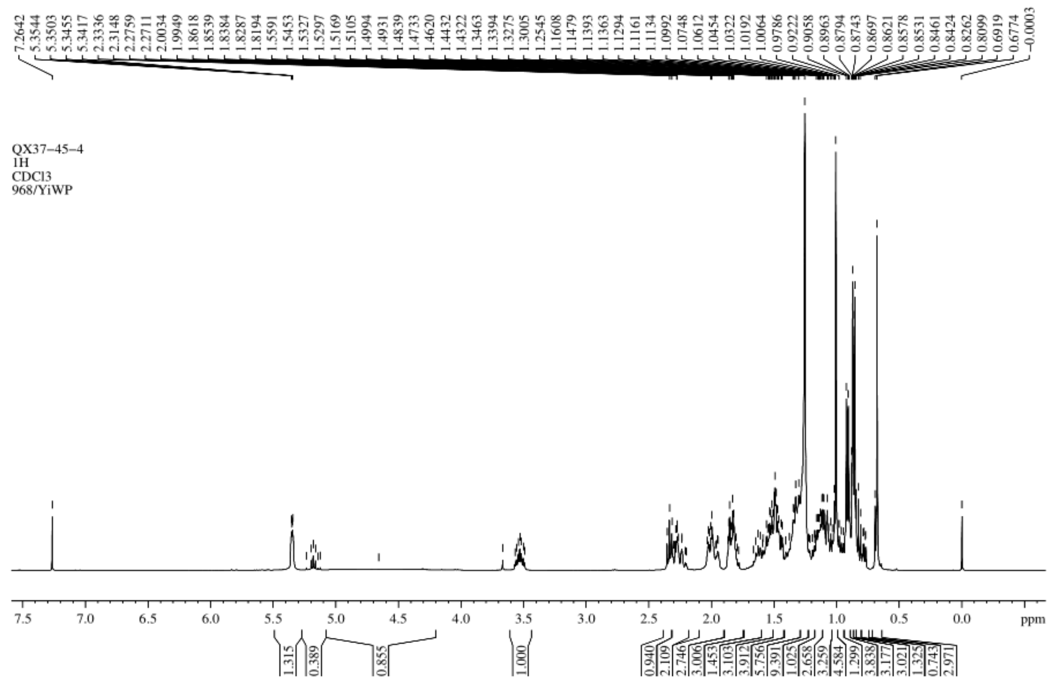

Figure S8. ^{1}H NMR spectrum of Compound 2 in $CDCl_3$ (400 MHz).

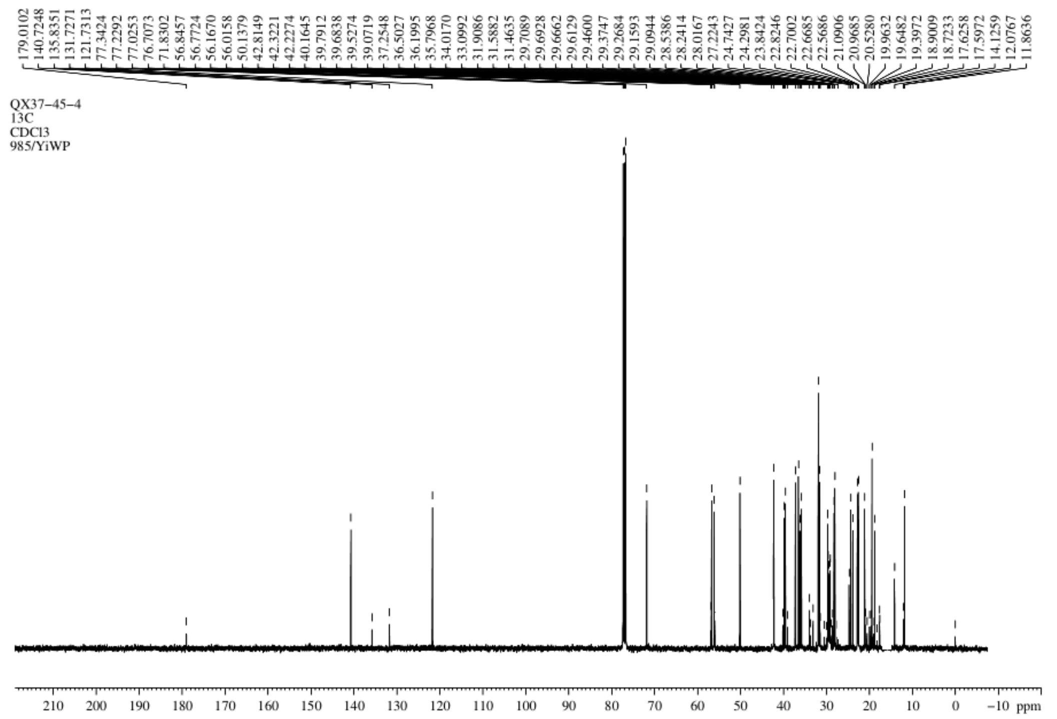

Figure S9. ^{13}C NMR spectrum of Compound 2 in $CDCl_3$ (400 MHz).

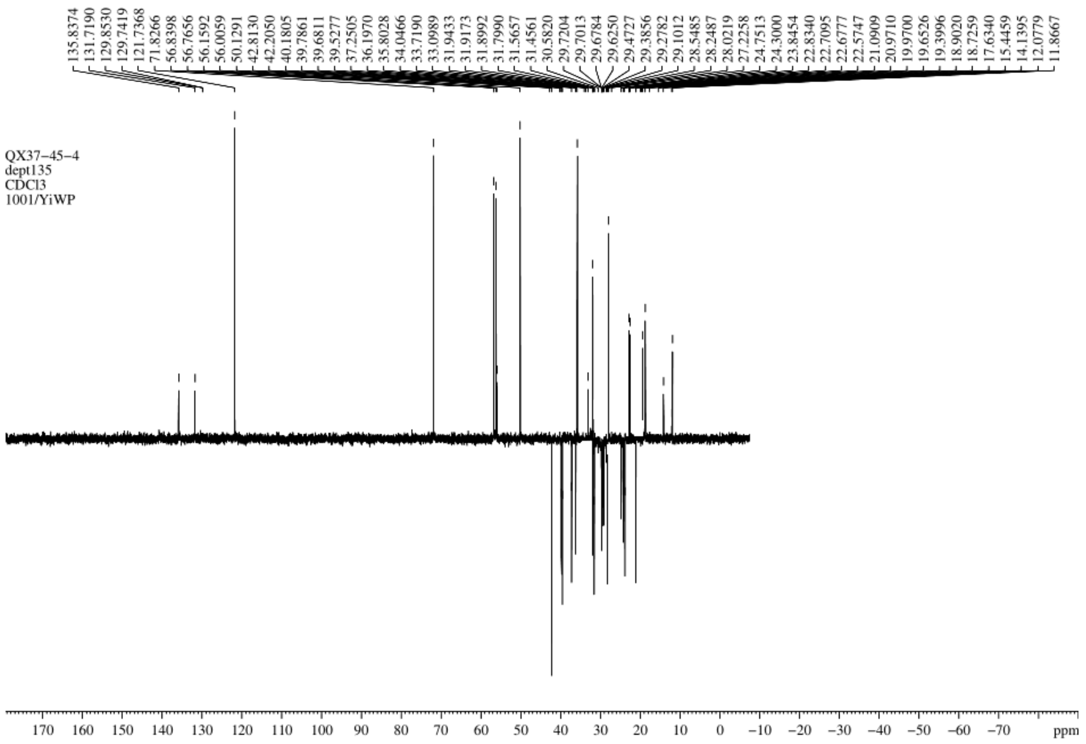

Figure S10. DEPT spectrum of Compound 2 in $CDCl_3$ (400 MHz).

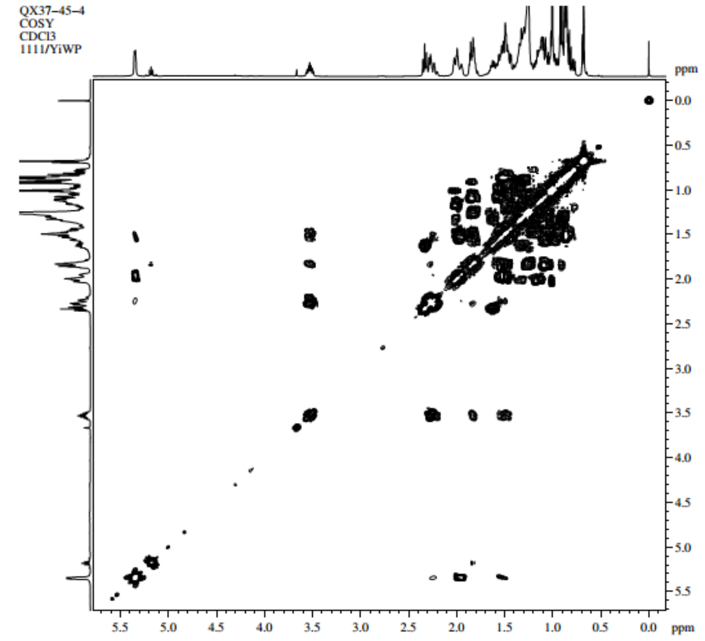

Figure S11. ^{1}H- ^{1}H COSY spectrum of Compound 2 in $CDCl_3$ (400 MHz).

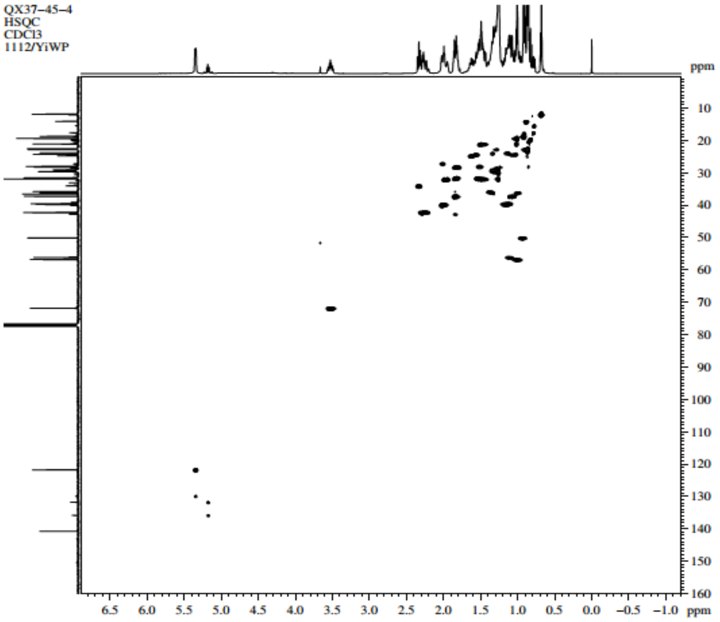

Figure S12. HSQC spectrum of Compound 2 in $CDCl_3$ (400 MHz).

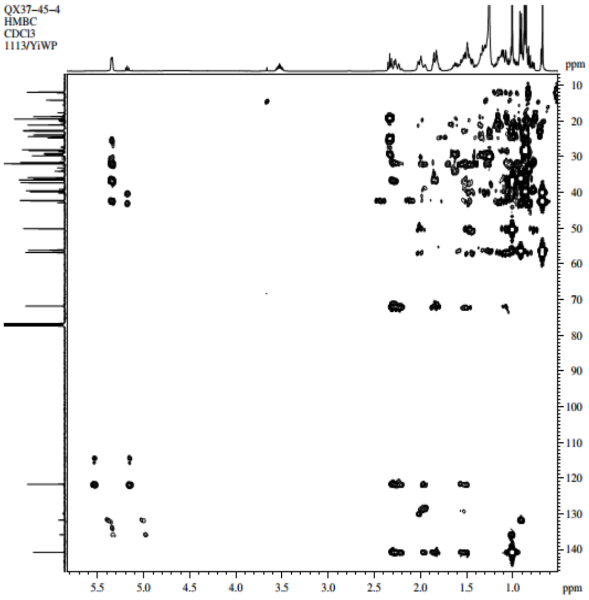

Figure S13. HMBC spectrum of Compound 2 in $CDCl_3$ (400 MHz).

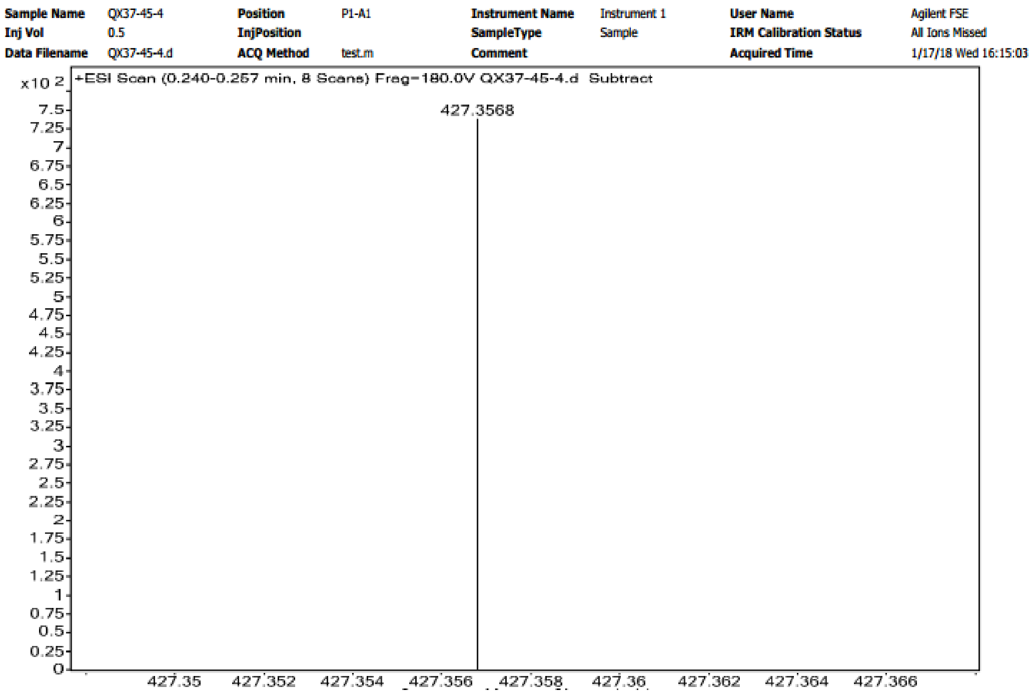

Figure S14.HR-ESIMS spectrum of Compound 2

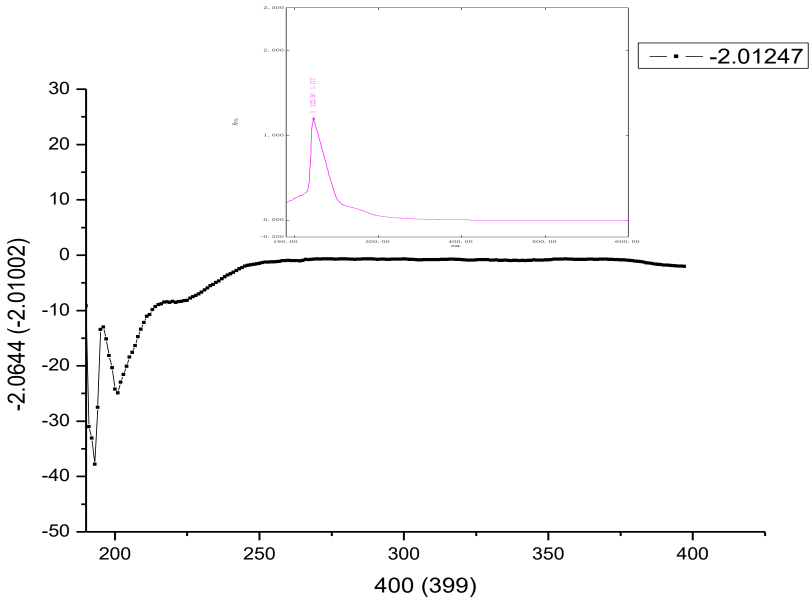

Figure S15 . Circular Dichromism spectra spectrum of Compound 2

Compound 3(QX3-2-7)**.**

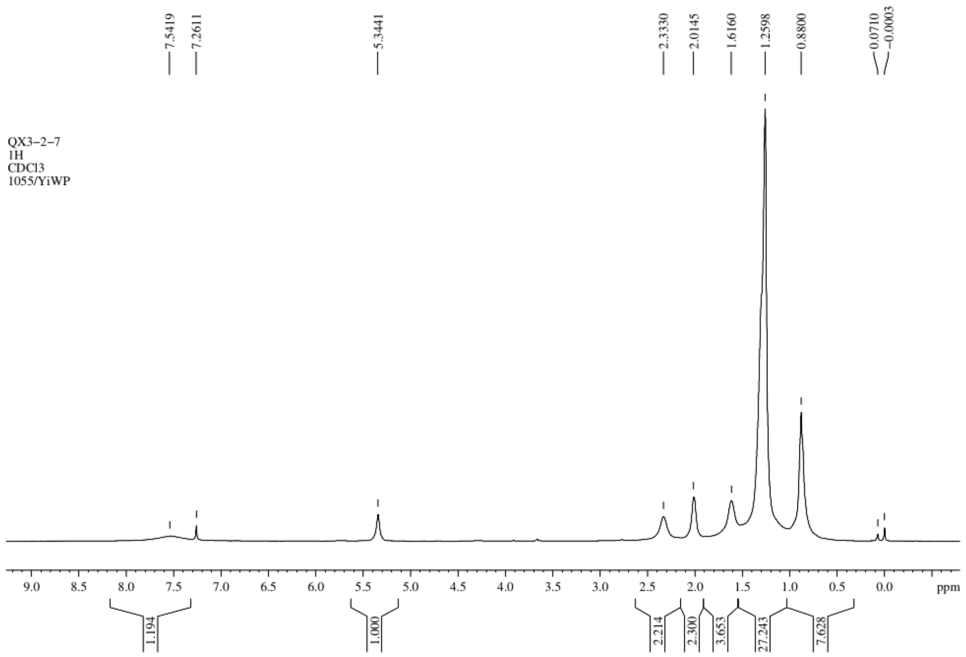

Figure S16. ^{1}H NMR spectrum of Compound 3 in $CDCl_3$ (400 MHz).

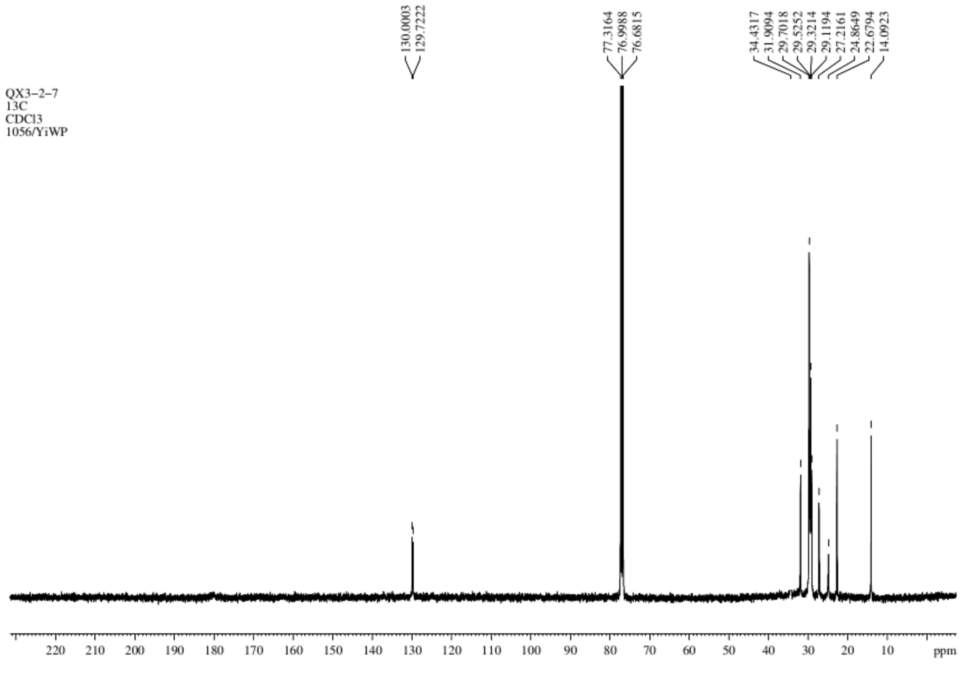

Figure S17. ^{13}C NMR spectrum of Compound 3 in $CDCl_3$ (400 MHz).

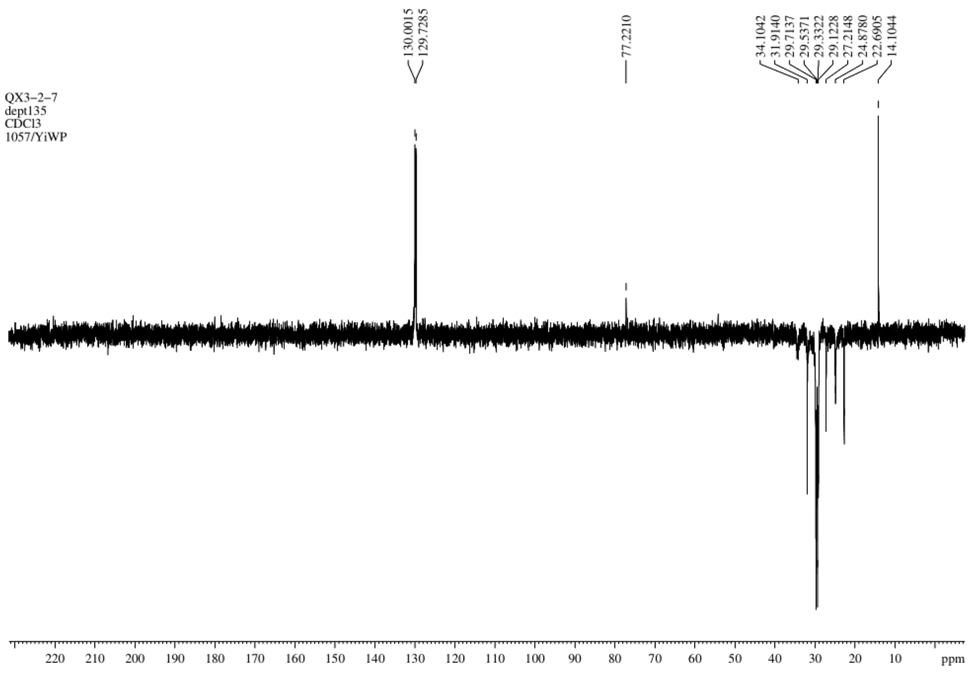

Figure S18. DEPT spectrum of Compound 3 in $CDCl_3$ (400 MHz).

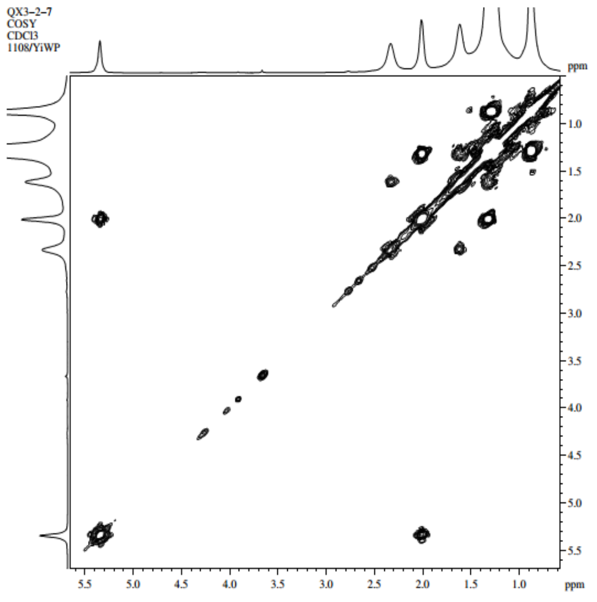

Figure S19. ^{1}H- ^{1}H COSY spectrum of Compound 3 in $CDCl_3$ (400 MHz)

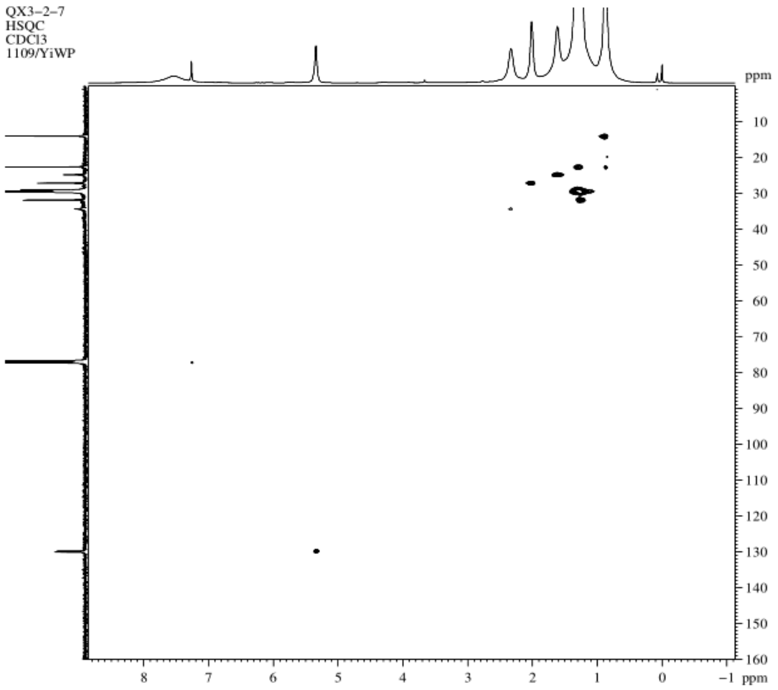

Figure S20. HSQC spectrum of Compound 3 in $CDCl_3$ (400 MHz).

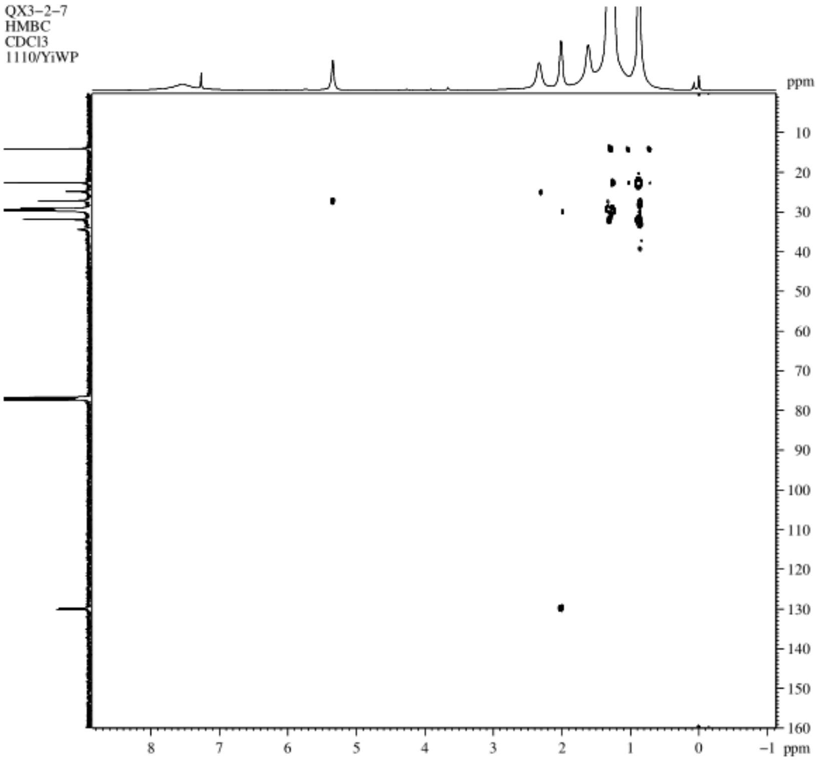

Figure S21. HMBC spectrum of Compound 3in $CDCl_3$ (400 MHz).

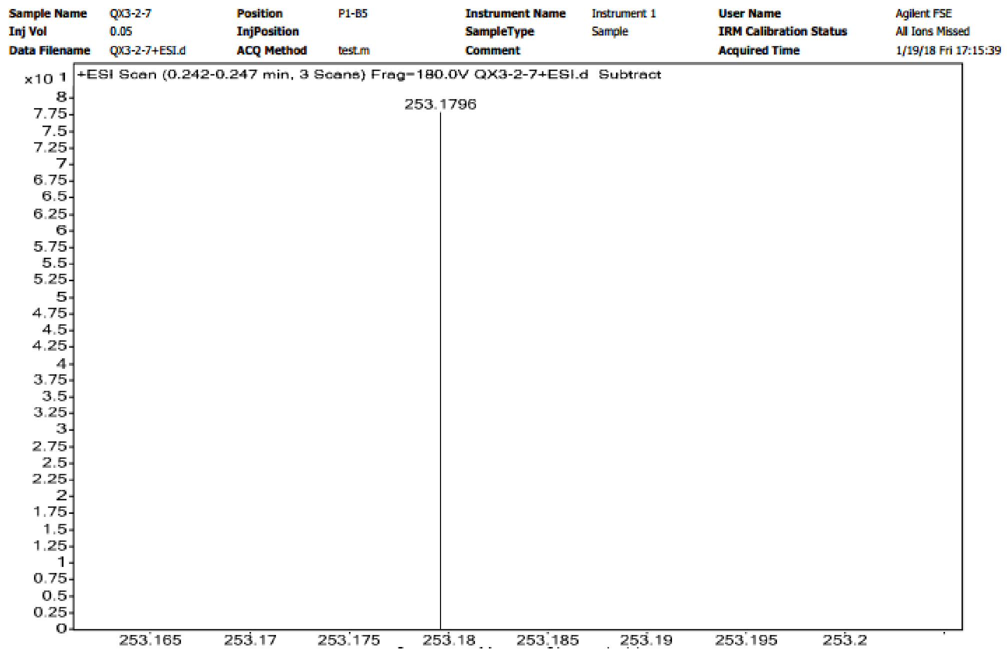

Figure S22. HR-ESIMS spectrum of Compound 3

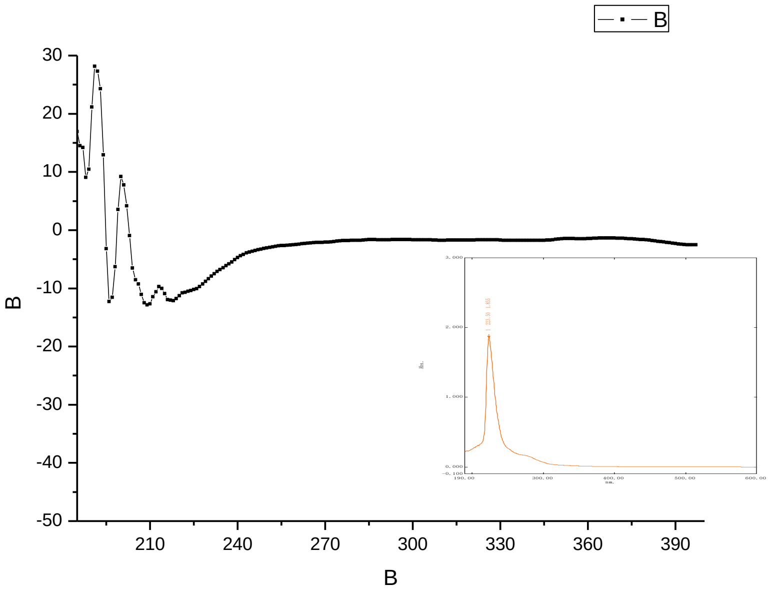

Figure S23 . Circular Dichromism spectra spectrum of Compound 3
